# Pickering Emulsion Stabilized by Tea Seed Cake Protein Nanoparticles as Lutein Carrier

**DOI:** 10.3390/foods11121712

**Published:** 2022-06-10

**Authors:** Li Liang, Junlong Zhu, Zhiyi Zhang, Yu Liu, Chaoting Wen, Xiaofang Liu, Jixian Zhang, Youdong Li, Ruijie Liu, Jiaoyan Ren, Qianchun Deng, Guoyan Liu, Xin Xu

**Affiliations:** 1 College of Food Science and Engineering, Yangzhou University, Yangzhou 225127, China; liangli0508@hotmail.com (L.L.); z17502110240@163.com (J.Z.); zhzhyi0928@163.com (Z.Z.); liuyu1998wang@163.com (Y.L.); chaoting@yzu.edu.cn (C.W.); liuxf@yzu.edu.cn (X.L.); zjx@yzu.edu.cn (J.Z.); liyoudonghh@163.com (Y.L.); yzufsff@163.com (G.L.); 2 National Engineering Research Center for Functional Food, Collaborative Innovation Center of Food Safety and Quality Control in Jiangsu Province, School of Food Science and Technology, Jiangnan University, Wuxi 214122, China; ruijieliu-2007@hotmail.com; 3 School of Food Science and Engineering, South China University of Technology, Guangzhou 510640, China; jyren@scut.edu.cn; 4 Hubei Key Laboratory of Lipid Chemistry and Nutrition, Key Laboratory of Oilseeds Processing, Ministry of Agriculture and Rural Affairs, Oil Crops Research Institute, Chinese Academy of Agricultural Sciences, Wuhan 430062, China; dengqianchun@caas.cn

**Keywords:** tea seed cake, protein, hydrothermal, nanoparticles, Pickering emulsion, lutein, delivery, in vitro digestion, bioaccessibility

## Abstract

To effectively deliver lutein, hydrothermally prepared tea seed cake protein nanoparticles (TSCPN) were used to fabricate Pickering emulsion, and the bioaccessibility of lutein encapsulated by Pickering emulsion and the conventional emulsion was evaluated in vitro. The results indicated that the average size and absolute value of zeta potential of TSCPN increased along with the increase in the protein concentration, and 2% protein concentration was adopted to prepare TSCPN. With the increase in the concentration of TSCPN, the size of Pickering emulsion decreased from 337.02 μm to 89.36 μm, and when the TSCPN concentration was greater than 0.6%, all emulsions exhibited good stability during the 14 days storage. Combined with the microstructure result, 1.2% TSCPN was used to stabilize Pickering emulsion. With the increase in ionic concentration (0–400 mM), the particle size of the emulsions increased while the absolute value of zeta potential decreased. TSCPN-based Pickering emulsion was superior to the conventional emulsion for both lutein encapsulation (96.6 ± 1.0% vs. 82.1 ± 1.4%) and bioaccessibility (56.0 ± 1.1% vs. 35.2 ± 1.2%). Thus, TSCPN-based Pickering emulsion in this study have the potential as an effective carrier for lutein.

## 1. Introduction

Lutein is a xanthophyll of the carotenoid family, and numerous research studies have revealed that it has antioxidative, anti-inflammatory, anti-cancer, oculo-protective, cardioprotective and immunomodulatory effects [1]. However, lutein cannot be synthesized by humans, and it can only be ingested from one’s diet [2], such as yellow corn, pepper, and egg yolks, etc. Lutein mainly relies on a passive diffusion pathway through the intestinal epithelial membrane to enter the human circulation, its hydrophobicity and environmental instability prevent it from being stably transported into the gut, resulting in limited bioaccessibility [3]. Therefore, it is necessary to develop lutein-encapsulated delivery systems to overcome these limitations.

The delivery systems that have been developed include emulsions, liposomes, nanoparticles and so on [4]. Due to the excellent stability, simple preparation process, and potential applications in the delivery of bioactive compounds, Pickering emulsions have attracted great attention in food, pharmaceuticals, and cosmetic fields. Pickering emulsions have an excellent stability to coalescence and even Ostwald ripening, mainly due to the formation of a flexible but robust physical colloidal barrier by solid particles at the liquid-liquid interface [5,6]. The low cost and environment-friendly processes, such as the hydrothermal method to make nanoparticles and one-step high-speed shearing at room temperature to make emulsion, make Pickering emulsion attractive for industry [7,8]. Pickering emulsions stabilized by starch, β-lactoglobulin, β-cyclodextrin nanoparticles, or complexed with polysaccharides, have been fabricated to prolong the storage time of lutein [9,10,11,12,13,14,15]; however, the bioaccessibility of lutein entrapped by Pickering emulsions has not been reported.

Increasing consumer demand for clean label, sustainable, and biocompatible products has made the search for plant-based particle stabilizers a hot topic in food research. The low solubility of plant proteins in both hydrophilic and hydrophobic media is consistent with the requirements of Pickering emulsion stabilizers [7,16]. Although soybean protein, peanut protein, etc., have been used as Pickering emulsion stabilizers [17,18], it is still necessary to develop more available proteins to find excellent stabilizers. Tea seed cake protein (TSCP) is extracted from the seed of tea tree (*Camellia sinensis* (L.) O, Kuntze), which is usually discarded or made into feed, causing a lot of waste. Our previous study revealed that TSCP had balanced amino acids composition and ordered structure [19], which are considered favorable factors for the formation of nanoparticles to stabilize Pickering emulsions [20]. Thus, it is necessary to further investigate whether nanoparticles fabricated from TSCP (TSCPN) can stabilize Pickering emulsions and deliver lutein effectively [21,22].

In this study, we first characterized the hydrothermally prepared TSCPN with different TSCP concentrations. Then, the TSCPN-stabilized Pickering emulsions were characterized, and the storage stability was continuously observed for 14 days. Finally, lutein was encapsulated by TSCPN-stabilized Pickering emulsion, and in vitro gastrointestinal digestion were performed to evaluate its bioaccessibility.

## 2. Materials and Methods

### 2.1. Materials

Tea seed was bought from Hubei, China, and the proximate composition was determined [19]. Nile red, Fluorescein isothiocyanate (FITC), and porcine pancreatic lipase (100–650 U/mg) were purchased from Sigma-Aldrich (St. Louis, MO, USA). Porcine pepsin (12 U/mg) and porcine pancreatin (40 U/mg) were obtained from Sunson (Beijing, China). Corn oil was obtained from a local grocery (Suguo, Yangzhou, China). All chemical reagents in this study were of analytical grade.

### 2.2. Preparation of TSCP

TSCP was prepared based on protein extraction methods previously optimized by our laboratory [19].

Tea saponins were removed firstly prior to protein extraction due to its bitter taste and haemolytic toxicity [23]. The TSC was crushed and dispersed in 90% ethanol, (1:10, *w*/*v*), stirred in a water bath at 30 °C for 2 h, and then centrifuged (L550 Cence, Changsha, Hunan, China) at 4000 rpm for 15 min. After removing the supernatant, the above operation was repeated until most saponins were removed. Vanillin-sulfuric acid method [24] was used to measure the content of tea saponin.

The processed TSC was dissolved in water (1:20, *w*/*v*), followed by adjusting to pH 10.5, incubated in water bath at 50 °C for 30 min, centrifuged at 4000 rpm for 30 min. The supernatant was collected and was adjusted to pH 3.6 using 1 M HCl, then the sample was centrifuged at 4000 rpm for 30 min. The precipitate was washed, dialyzed for 24 h, and freeze-dried (CTFD-18S, CREATRUST, Qingdao, China). The freeze-dried protein sample was kept at −20 °C before use.

### 2.3. Preparation and Characteristics of TSCPN

The TSCPN preparation method was slightly modified from Ren et al. [25]. In brief, TSCPN was prepared using the hydrothermal method. TSCP was dissolved in RO water and adjusted to pH 7, stirred in a water bath at 90 °C for 1.5 h, centrifuged with 4000 rpm to remove insoluble particles, and then the supernatant was stored in a refrigerator at 4 °C. In order to explore the effects of TSCP concentration on TSCPN features, different concentrations (0.5%, 1%, 2%, 3%, 4%) of TSCPN were prepared according to the above method.

The measurement methods for particle size and zeta potential were according to Liang et al. [26]. Specifically, after 100-fold dilution of TSCPN, the particle size and zeta potential of TSCPN were analyzed (Zetasizer Nano ZS, Malvern, UK), and the refractive indices of the dispersant and material were set to 1.330 and 1.470, respectively. All tests were repeated at least three times.

After lyophilization, the microstructure of TSCPN was characterized using field emission scanning electron microscopy (s-4800, HITACHI, Tokyo, Japan).

### 2.4. Preparation and Characteristics of TSCPN-Stabilized Pickering Emulsion

We aimed to investigate the effect of TSCPN concentration on emulsion. The Pickering emulsion was prepared using oil fractions (φ = 0.4 *v*/*v*) and different concentrations of TSCPN [27]. In brief, 4 mL of corn oil was added to 6 mL of TSCPN solution with different concentrations (0.3%, 0.6%, 1.2%, 1.8%, and 2.4%, *w*/*v*), then, the corn oil phase and TSCPN were mixed by high-speed homogenizer (T18, IKA, Staufen, Germany) at 20,000 rpm at ice-water bath for 3 min. The prepared emulsion was stored at 4 °C in the dark.

This type of TSCPN-stabilized Pickering emulsion was determined according to the drop test method [28]. In brief, emulsion was added to RO water and corn oil, respectively. If the droplets of emulsions agglomerated in the water and dispersed in the corn oil, it was considered as water-in-oil emulsions, otherwise, it was regarded as oil-in-water emulsions.

The method for determining the average particle size of the emulsion is the same as that for the nanoparticles described above. All tests were repeated at least five times. The microstructures of the emulsions were observed by laser scanning confocal microscope (LSM 880NLO, Carl Zeiss AG, Oberkochen, Germany). Before observation, Lipid and TSCPN were stained by 2–5 drops 1 mg/mL Nile red and 1 mg/mL FITC, respectively. The excitation and emission spectrum were 543 and 605 nm for Nile red, whereas they were 488 and 515 nm for FITC. In this case, Nile red fluoresces red, whereas FITC fluoresces green.

In addition, in order to investigate the influence of ionic strength. NaCl (final concentration 0–0.4 M) was added to Pickering emulsion, the preparation and characterization of the emulsions were carried out according to the above procedure.

### 2.5. Storage Stability

In order to explore the storage stability of Pickering emulsion at 4 °C, creaming index (*CI*) was evaluated. The of *CI* value was calculated as follows.

$$CI\%=\frac{H_{s}}{H_{t}}\times 100$$

where *H_s_* and *H_t_* represent the height of the serum and all phases, respectively.

### 2.6. In Vitro Digestion Model

The ability of TSCPN-stabilized Pickering emulsions to deliver lutein was evaluated by measuring the release rate of lutein at different stages and the bioaccessibility after digestion in an in vitro digestion model. In addition, particle size and microscopic changes during digestion were measured as described in Section 2.4.

The in vitro digestion model is modified from the method of Liang et al. [26]. First, gastric and intestinal fluid storage solutions were prepared. Gastric stock solution was made up of 35.16 mM NaCl and 227.58 mM HCl; intestinal fluid stock solution was made up of 3.75 M NaCl and 247.53 mM CaCl. Bile salts had to be prepared 12 h in advance, and 0.5352 g of pig bile salts were dissolved in 10 mL of neutral phosphate buffer.

Due to the short retention time of the emulsion in the oral cavity, the influence of oral phase was not considered. Before entering the gastric phase, the emulsion was diluted to a 1% fat concentration for optimal digestion [29].

Gastric phase. We took 20 mL of gastric juice stock solution and incubated it at 37 °C, then mixed 20 mL of the diluted emulsion with 20 mL of gastric working solution, adjusted to pH 2.5, added 1.33 g pepsin and adjusted to pH 2.5, kept the incubation in a constant temperature shaker at 37 °C, 100 rpm culture for 2 h.

Small Intestinal Phase. We took 30 mL of gastric chyme, incubated it at 37 °C to adjust pH 7.0, then added 1.5 mL of intestinal fluid storage solution and 3.5 mL of bile salts, and adjusted to pH 7.0 again. We added 0.6 g trypsin and 0.4 g trypsin. 37 °C, 100 rpm constant temperature shaker for 2 h.

Lutein was extracted from chyme with 3 volumes of ethanol and acetone = 1:1 (*v*/*v*), and the mixture was shaken for 1 min and centrifuged at 5000 rpm for 5 min. The supernatant was collected to measure the absorbance at 450 nm. The release rate of lutein was calculated by the following equation:

$$RR\left(\%\right)=\frac{C_{F}}{C_{I}}\times 100\%$$

where *C_F_* is the concentration of free lutein, and *C_I_* is the theoretical concentration of lutein at different stages.

Bioaccessibility was calculated according to the report of E Fernández-García et al. [30]. After gastrointestinal simulation, the upper micelle was collected after centrifugation (8000 rpm, 30 min), and lutein was measured as above. Bioaccessibility is calculated according to the following equation:

$$a\left(\%\right)=\frac{C_{M}}{C_{R}}\times 100\%$$

where *C_M_* is the lutein concentration in the micelle phase and *C_R_* is the concentration of lutein in the entire intestinal phase.

The degree of protein hydrolysis was measured by neutral formaldehyde titration method [31]. In short, 10 mL of chyme was added to 5 drops of 30% hydrogen peroxide, adjusted to pH 7.5, 15 mL of neutral methanol was added, and titrated with 0.15 M NaOH after 1 min of reaction, and then the consumption volume of NaOH was recorded. The undigested TSCPN was set as blank control. The degree of hydrolysis can be calculated by the following formula:

$$C\left(\%\right)=\frac{(V_{1}-V_{0})\times V_{w}\times C_{NaOH}\times 14.01\times 6.25}{1000\times M}$$

where *V*_1_ is the volume of NaOH solution consumed by digesta(mL); *V*_0_ is the volume of NaOH solution consumed by blank (mL); *V_w_* is the volume of water for ingredients (mL); *C_NaOH_* is the concentration of NaOH solution for titration (0.15 M); 14.01 is the molar mass of nitrogen; 6.25 is the coefficient of nitrogen conversion to protein; *M* is the mass of TSCP.

### 2.7. Statistical Analysis

Triplicate tests were performed for each sample. The data were handled as the mean values ± standard deviation. The differences among the tests were evaluated by one-way ANOVA using Origin 2021 software (*p* < 0.05).

## 3. Result and Discussion

### 3.1. Effect of TSCP Concentrations on the Properties of TSCPN

#### 3.1.1. Effect of TSCP Concentrations on the Particle Size and Zeta Potential of TSCPN

**Figure 1:**
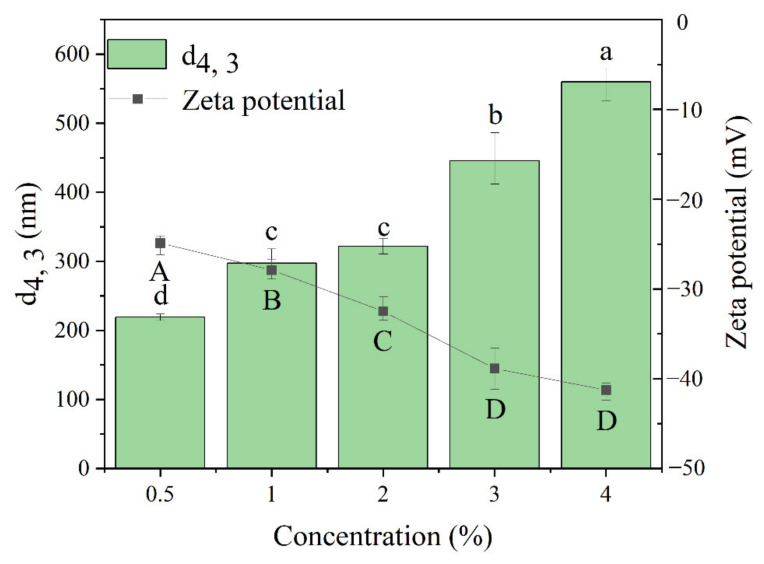
Effect of tee seed cake protein (TSCP) concentrations on the particle size (volume mean diameter d_4__, 3_ histogram) and zeta potential (line) of nanoparticles fabricated from TSCP (TSCPN). Different lowercase letters indicated significant differences of particle size between treatments (*p* < 0.05), and different uppercase letters indicated significant differences of zeta potential between treatments (*p* < 0.05).

The properties of nanoparticles determine their ability to stabilize Pickering emulsions. Among them, particle size and potential are two important indicators. Specifically, the smaller the particle size, the faster the nanoparticles adsorb to the oil-water interface [32]. In addition, the electrostatic repulsion plays a vital role in the stabilization of particles against aggregation [33]. It has been reported that protein concentration has a significant effect on the properties of hydrothermally prepared protein nanoparticles [34]. As shown in Figure 1 (histogram), the average particle size of the TSCPN increased significantly with the increase in TSCP concentration (except for 1.0–2.0%), which may be related to the increased chance of particle-to-particle interactions [35]. Similar results were found in the study of soy protein nanoparticles by Fu et al. [34]. It is worth noting that, the suitable particle size of protein nanoparticles developed to stabilize Pickering emulsions is mostly in the range of 100–350 μm [36]. In addition, the particle size of TSCPN prepared with 0.5–2.0% concentration of TSCP was within this range. Moreover, it can be seen from Figure 1 (line graph) that with the increase in TSCP concentration, the absolute value of zeta potential of TSCPN increased significantly. The higher zeta potential means that the nanoparticles have greater electrostatic repulsion, which is beneficial to the stabilization of the water–oil interface in the emulsions [37]. Under the premise of suitable particle size, TSCPN prepared with 2.0% TSCP had the high absolute value of zeta potential (−32.5 ± 1.6 mV). Therefore, the TSCPN prepared by TSCP with a concentration of 2.0% had the potential to stabilize the Pickering emulsion.

#### 3.1.2. The Microstructure of TSCP and TSCPN

**Figure 2:**
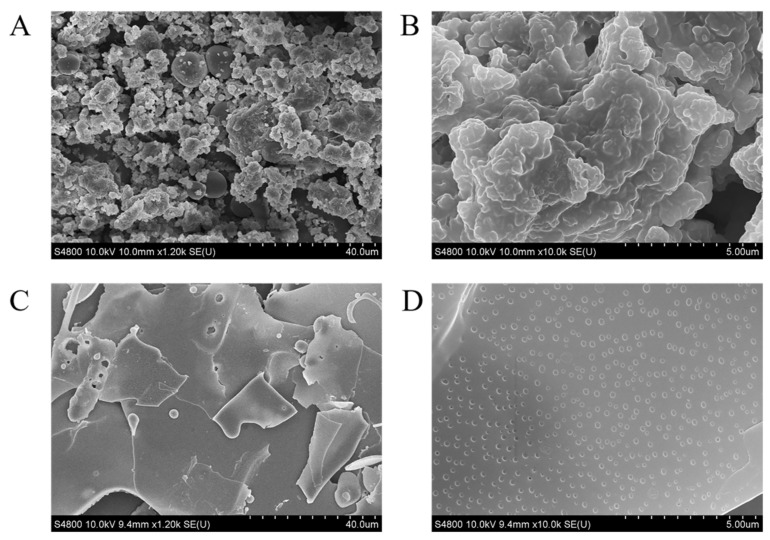
SEM image of TSCP (**A**,**B**) and TSCPN (**C**,**D**). The magnifications of (**A**,**C** and **B**,**D**) are 1200 and 10,000 times, respectively.

The morphology of solid nanoparticles can affect their ability to stabilize Pickering emulsions, specifically the particles’ shape governs their behavior at the interface [38]. According to the above results, the microstructure of TSCPN prepared with a concentration of 2.0% TSCP was observed. After hydrothermal treatment, the porous spherical shape of TSCP has been changed significantly, and a thin plate structure composed of microspheres of TSCPN was formed (Figure 2). The non-deformable nanoparticle structure of TSCPN had the characteristics of a large specific surface area, thus they can adsorb to the water–oil interface quickly, which was necessary to stabilize Pickering emulsions [39].

### 3.2. Characteristics of TSCPN-Stabilized PICKERING Emulsion

#### 3.2.1. Type of TSCPN-Stabilized Pickering Emulsion

The TSCPN-stabilized Pickering emulsion was added dropwise to water and oil, respectively, and the type of emulsion was determined according to its dispersion and aggregation state. According to titration experiments, TSPN-stabilized Pickering emulsions aggregated in oil and diffused in water, which indicated that the TSPN-stabilized Pickering emulsion was an O/W emulsion.

#### 3.2.2. Effect of TSCPN Concentrations on the Particle Size and Micromorphology of Pickering Emulsions

**Figure 3:**
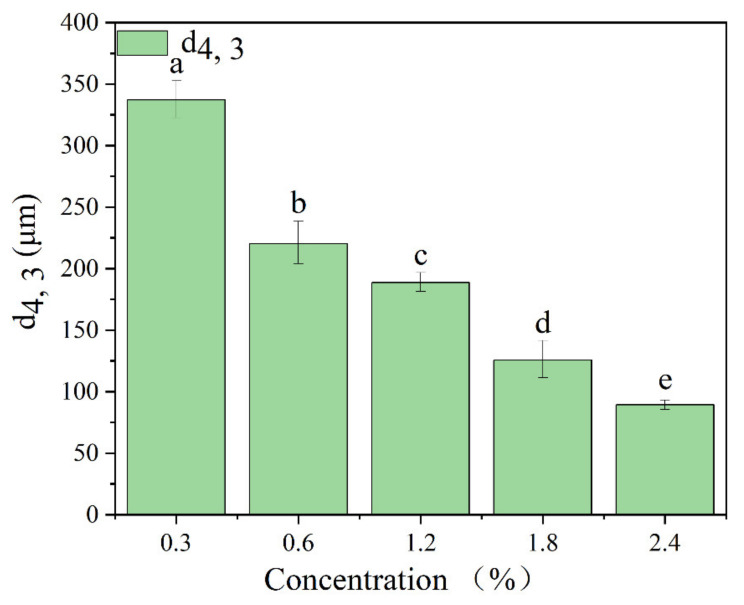
Volume mean diameter (d4, 3) of Pickering emulsions stabilized by different concentrations of TSCPN. Different lowercase letters indicated significant differences (*p* < 0.05).

The stability of Pickering emulsions is significantly affected by the concentration of solid particles [40]. In addition, the stability of the emulsion can be reflected by particle size, because the smaller the particle size, the less aggregation. Figure 3 showed the average particle size of Pickering emulsions stabilized by different concentrations of TSCPN at a constant oil phase fraction of 0.4. The average particle size of Pickering emulsions decreased significantly with increasing TSCPN concentration. This may be due to the fact that more TSCPN adsorbed to the water–oil interface, resulting in a larger specific surface area of the emulsion [41]. This phenomenon was also observed in Pickering emulsions stabilized by soy protein nanoparticles [34] and rapeseed protein nanogels [42].

**Figure 4:**
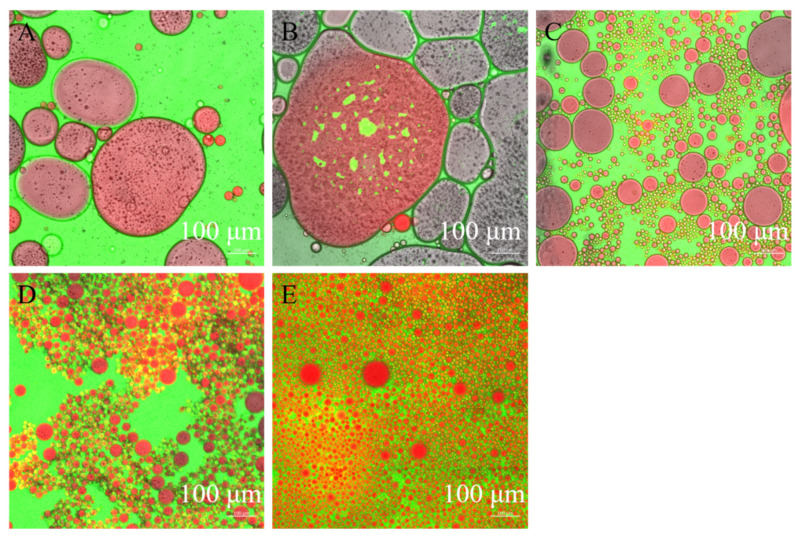
CLSM images of Pickering emulsions stabilized by different concentrations of TSCPN. (**A**), 0.3%; (**B**), 0.6%; (**C**), 1.2%; (**D**), 1.8%; (**E**), 2.4%. Oil was dyed red, and protein was dyed green.

CLSM images can intuitively reflect the behavior of nanoparticles at the water–oil interface. The microstructure of Pickering emulsions stabilized by different concentrations of TSCPN (Figure 4) presented consistent results with their particle sizes (Figure 3). When the concentration of TSCPN was low (0.3–0.6%), there was not enough TSCPN to form a complete physical barrier at the water–oil interface, and lots of oil droplets agglomerated. When the concentration of TSCPN increased to 1.2–2.4%, the oil droplets size gradually decreased and the distribution became uniform, which was attributed to more TSCPN adsorbed to the water–oil interface, and it formed a dense physical barrier. In addition, higher concentrations of TSCPN increased the contact between the droplets, resulting in greater electrostatic repulsion, which also contributed to the stability of Pickering emulsions [43]. Combining the average emulsion size and the CLSM image, it can infer that 1.2–2.4% TSCPN can stabilize the Pickering emulsion with an oil phase of 0.4.

#### 3.2.3. Effect of TSCPN Concentrations on the Storage Stability of Pickering Emulsions

**Figure 5:**
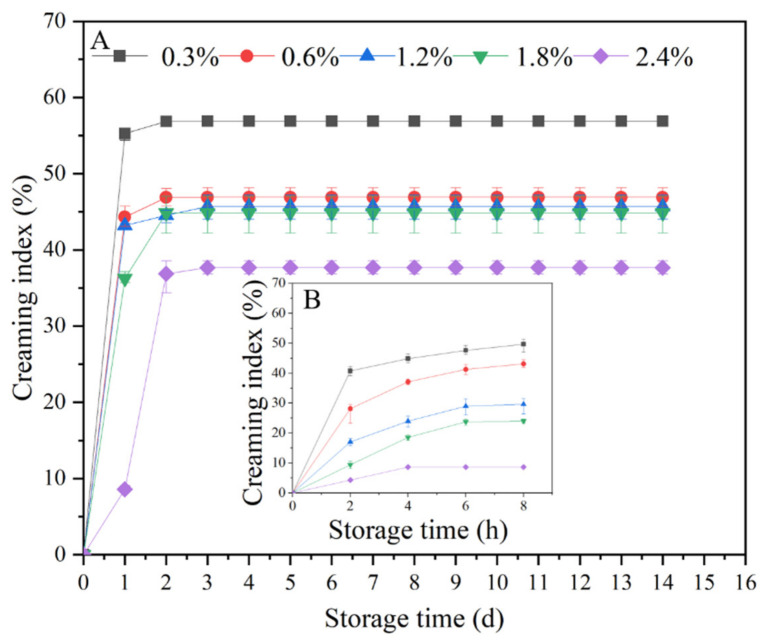
Changes in CI of Pickering emulsions stabilized by different concentrations of TSCPN over a storage period of 14 days (**A**) and within 8 h after preparation (**B**).

Good storage stability is a prerequisite for the use of emulsions in the food industry [44]. CI can reflect the aggregation and stratification of the emulsion, and then evaluate the storage stability of the emulsion, which has been used in the evaluation of various emulsions [42,45]. Within 8 h after fresh preparation, CI and its growth rate of the TSCPN-stabilized Pickering emulsion decreased significantly with increasing concentration of TSCPN (Figure 5B). This may be due to the smaller average size of Pickering emulsions stabilized by higher concentrations of TSCPN (Figure 3), which is helpful to slow down the aggregation of the emulsions. The same phenomenon was also observed in long-term storage over 14 days (Figure 5A), and CI of all Pickering emulsions entered a plateau at the third day of storage. This process has also been reported in other Pickering emulsions [42,46]. This suggests that the concentration of TSCPN is important not only to the formation, but also to the stabilization of Pickering emulsions on storage. A TSCPN concentration of 1.2% was chosen for the following experiments.

#### 3.2.4. Effect of Ionic Strength on the Particle Size and Zeta Potential of Pickering Emulsions

**Figure 6:**
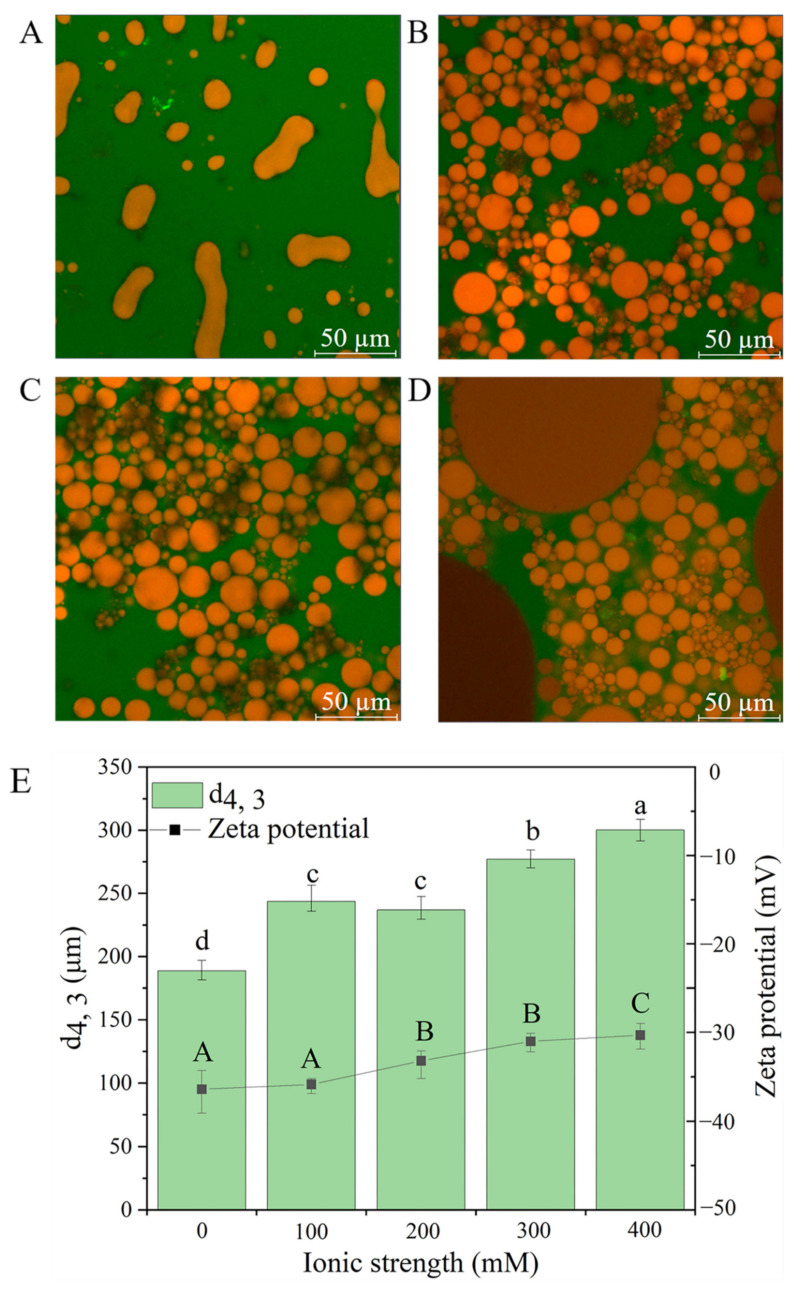
CLSM images (**A**–**D**) of the effect of different ion concentrations (100–400 mM) on the Pickering emulsion. Effect of ionic strength on particle size d_4__, 3_ (histogram in (**E**)) and zeta potential (line in **E**) of Pickering emulsion. Different lowercase letters indicate significant difference of particle size between treatments (*p* < 0.05), and different uppercase letters indicated significant differences of zeta potential between treatments (*p* < 0.05).

Salt ions are ubiquitous in food matrices, and many studies have shown that ionic strength has a significant effect on Pickering emulsions [47,48]. Thus, it is necessary to investigate the effect of ionic strength on TSCPN-stabilized Pickering emulsions. Figure 6 (histogram in E) showed that the particle size of the emulsions increased along with the increase in ionic concentration, and the microstructural image also indicated that the droplets aggregation had occurred (Figure 6A–D). The result might be attributed to the electrostatic screening effect of ions, which can cause the reduction in electrostatic repulsion and aggregation of droplets. The significant decrease in the absolute value of the zeta potential of Pickering emulsions confirmed this hypothesis (Figure 6 line in E). It was worth noting that a slight decrease in droplet size occurred at 200 mM ionic strength. Although the increase in ionic strength negatively impacted the stability of Pickering emulsions overall, appropriate ionic strength will reduce the electrostatic repulsion of nanoparticles, and then free nanoparticles adsorb on the water–oil interface. The result could be evidenced by their microstructural images as well. The droplet exhibited irregular shape at low 100 mM of ionic concentration, and the droplets reverted to spherical shape at higher concentrations of ionic strength. Wang et al. also observed similar phenomena in the rapeseed-protein-nanogel-stabilized Pickering emulsion [42].

### 3.3. Encapsulation and Delivery of Lutein: An In Vitro Digestion Model

**Figure 7:**
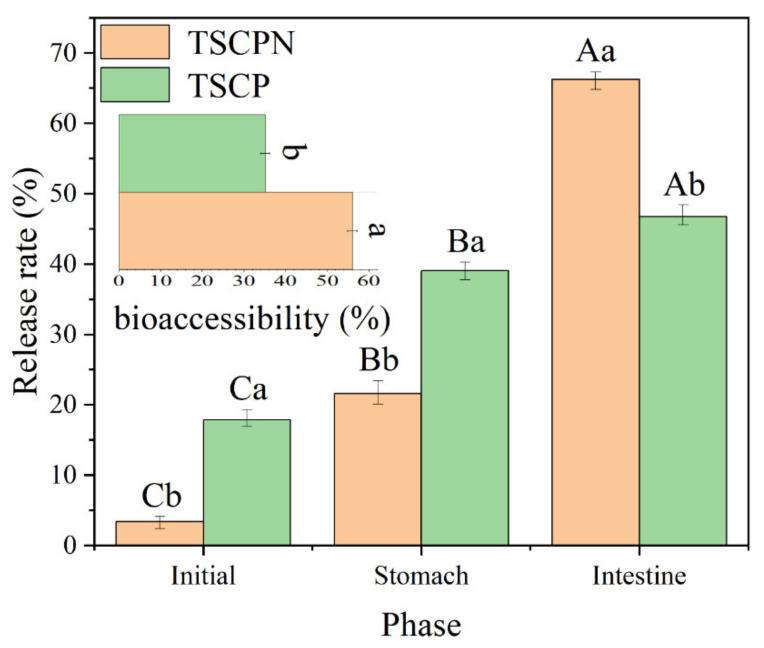
Lutein release rates (vertical bar graphs) of TSCPN-stabilized Pickering emulsions and TSCP-stabilized emulsions at initial stage, post-gastric, and post-intestinal; TSCPN-stabilized Pickerings and TSCP-stabilized post-intestinal Lutein bioaccessibility of emulsion delivery (horizontal bar graph). Different lowercase letters indicated significant differences between TSCPN-stabilized Pickering emulsion and TSCP-stabilized emulsion (*p* < 0.05), different capital letters indicate significant difference among different digestion phases (*p* < 0.05).

The lutein delivery capacity of TSCPN-stabilized Pickering emulsion was evaluated in a simulated gastrointestinal digestion system, and TSCP-stabilized emulsion was prepared as a control. It can be seen from Figure 7 that there is 3.4 ± 1.0% and 17.9 ± 1.4% of free lutein initially in TSCPN-stabilized Pickering emulsion and TSCP-stabilized emulsion, respectively. The significantly higher encapsulation rate of Pickering emulsion may be due to the fact that TSCPN could quickly adsorb to the water–oil interface and form a dense physical barrier. Compared with the currently developed lutein delivery vehicles, such as nanocapsules with chitosan, nano lipid carriers with ω-3 fatty acid, etc., the TSCPN-stabilized Pickering emulsion has a higher encapsulation rate [1].

**Figure 8:**
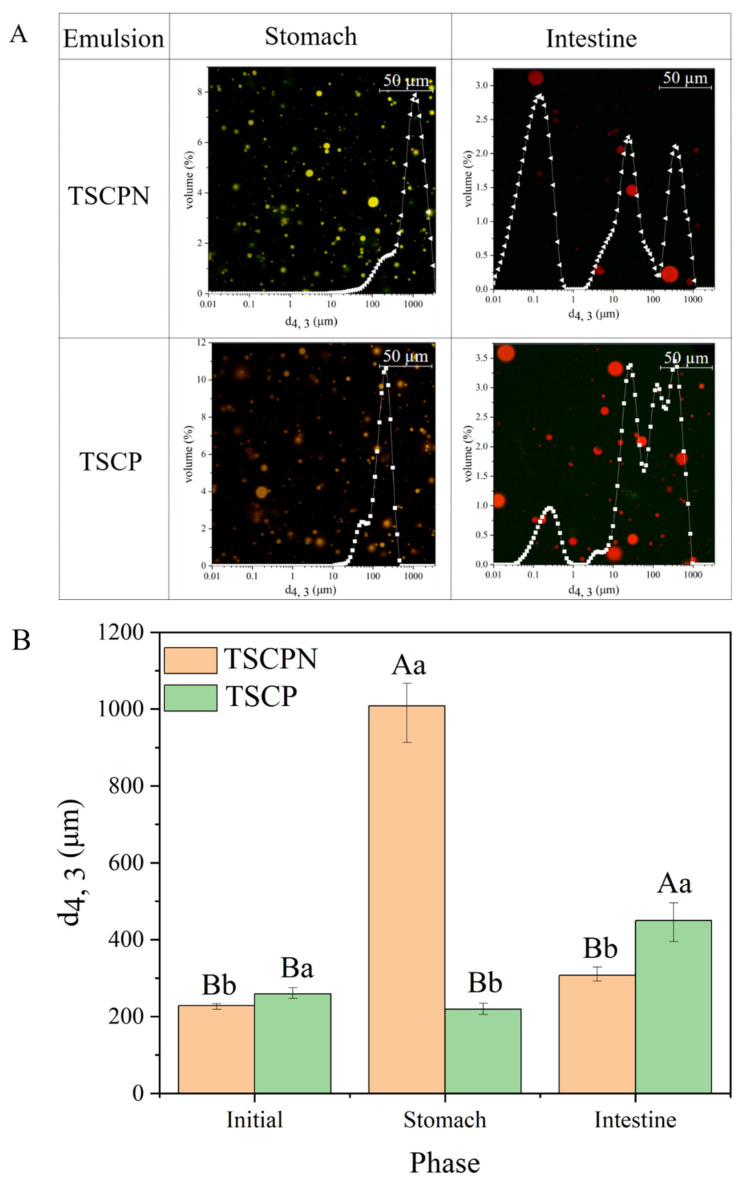
CLSM images, particle size distribution (**A**) and particle size (d4, 3) (**B**) of TSCPN-stabilized Pickering emulsion and TSCP-stabilized emulsion at different digestion periods. Different lowercase letters indicate significant differences between TSCPN-stabilized Pickering emulsions and TSCP-stabilized emulsions (*p* < 0.05), and different uppercase letters indicate significant differences at different digestion stages (*p* < 0.05).

After gastric digestion, 21.6 ± 1.8% and 39.1 ± 1.2% of lutein was released from the TSCPN-stabilized Pickering emulsion and TSCP-stabilized emulsion, respectively. Compared with the initial emulsion, more lutein was released from TSCP-stabilized emulsion than from TSCPN-stabilized Pickering emulsion. The result may be attributed to the different aggregation state of the droplets after gastric digestion, which can be confirmed from their average droplet size (Figure 8B). Compared with the initial droplet size, the droplet size of the TSCPN-stabilized Pickering emulsion was significantly increased, indicating more aggregation, and thus possibly inhibiting the release of lutein. The same phenomenon was also observed in the zein/soluble polysaccharide composite nanoparticle system [49].

Compared with the stomach phase, the cumulative release rate of lutein in both emulsions increased significantly after intestinal digestion. Although the release rate of lutein from TSCP-stabilized emulsions in the gastric phase was higher than that of TSCPN-stabilized Pickering emulsions, both the release rate and the bioaccessibility of lutein after intestinal digestion were significantly higher in the latter than in the former emulsion, indicating that TSCPN-stabilized Pickering emulsion has better delivery efficiency than the TSCP-stabilized emulsion. The difference of release rate of lutein may be due to the difference of the hydrolysis degree of protein in the interface. We verified the assumption by measuring the hydrolysis degree of protein after intestinal digestion in both emulsions, and the hydrolysis degree of protein in both emulsion for TSCPN and TSCP was 56.80 ± 2.55%and 27.89 ± 2.59%, respectively. In addition, the particle size measurement of the micellar phase found that the size of the micelles obtained from the TSCPN-stabilized Pickering emulsion was 279.81 ± 21.08 nm, whereas the size of the micelles obtained from the TSCPN-stabilized emulsion was 306.41 ± 17.58 nm. Studies have demonstrated that a smaller micellar phase can increase the bioaccessibility of lutein [50]. There are no studies evaluating Pickering emulsion delivery of lutein in vitro, so comparisons can only be made with other types of delivery systems. Yu et al. developed the composite nanoparticles from gum Arabic and carboxymethylcellulose-modified stauntonia brachyyanthera seed albumin for lutein delivery, and this system can increase the bioaccessibility of lutein to 46.8% [51]. Zhang et al. utilized glycosylated zein as a delivery vehicle for lutein and obtained a bioaccessibility of about 30% [52]. Compared with these developed carriers, TSCPN-stabilized Pickering emulsions exhibited better lutein delivery capacity during digestion in vitro.

## 4. Conclusions

A novel lutein delivery vehicle was prepared using TSCPN-stabilized Pickering emulsion. The particle size of TSCPN was affected by the concentration of TSCP, but the average particle size is between 321.55 ± 11.05–548.40 ± 8.7 nm. Pickering emulsion stabilized by 1.2% TSCPN showed good storage stability during the 14 days storage period. In addition, TSCPN-stabilized Pickering emulsion could significantly improve the bioaccessibility of lutein (56.0 ± 1.1%) in vitro, which was attributed to the high degree of hydrolysis of TSCPN. Therefore, the Pickering emulsion prepared in this study can be used as a good carrier to improve the bioaccessibility of lutein in beverages, salad dressing, etc.

## Data Availability

Data are contained within the article.
